# Femoral head wear and metallosis caused by damaged titanium porous coating after primary metal-on-polyethylene total hip arthroplasty: a case report

**DOI:** 10.3325/cmj.2018.59.253

**Published:** 2018-10

**Authors:** Domagoj Delimar, Ivan Bohaček, Damjan Dimnjaković, Dalibor Viderščak, Zdravko Schauperl

**Affiliations:** 1 Department of Orthopedic Surgery, University of Zagreb School of Medicine, Zagreb, Croatia; 2 Department of Orthopedic Surgery, University Hospital Center Zagreb, University of Zagreb School of Medicine, Zagreb, Croatia; 3 Department of Materials, Faculty of Mechanical Engineering and Naval Architecture, University of Zagreb, Zagreb, Croatia

## Abstract

Excessive metal femoral head wear has been described only as revision surgery complication after primary ceramic-on-ceramic total hip arthroplasty (THA). Here, we present the first case of metal femoral head wear after primary metal-on-polyethylene THA. A 56-year-old woman was referred to our outpatient clinic 17 years after primary right-sided THA, experiencing pain and decreased right hip range of motion. Radiographic examination revealed acetabular cup dislocation, eccentric femoral head wear, damaged titanium porous coating of femoral stem, metallosis, and pseudotumor formation. Endoprosthetic components were extracted, but further reconstruction was impossible due to presence of large acetabular bone defect. Macro- and micro-structure of extracted components were analyzed. Acetabular liner surface was damaged, with scratches, indentations, and embedded metal debris particles present on the entire inner surface. Analysis of metal debris by energy-dispersive spectroscopy showed that it consisted of titanium and stainless-steel particles. Femoral head was gravely worn and elliptically shaped, with abrasive wear visible under scanning electron microscope. No signs of trunnionosis at head/neck junction were observed. Microstructure of femoral head material was homogeneous austenitic, with microhardness of 145 HV 0.2, which is lower than previously described titanium hardness. In conclusion, detached titanium porous coating of femoral stem can cause stainless-steel femoral head wear in primary metal-on-polyethylene THA. As soon as such detachment becomes evident, revision surgery should be considered to prevent devastating complications.

The complications of total hip arthroplasty (THA), such as adverse reaction to metal debris, corrosion, and pseudotumor formation, are no longer reserved exclusively for metal-on-metal but can also occur in metal-on-polyethylene implants (1). These complications are mostly caused by tribocorrosion, a process that takes place at the head/neck and neck/stem junction and depends on implant modularity (2). Excessive metal femoral head wear has been described so far only as a complication of revision surgery after primary ceramic-on-ceramic THA (3). Here, we present the first case of metal femoral head wear after primary THA using metal-on-polyethylene bearing surfaces.

## Case report

A 56-year-old woman was referred to our outpatient clinic in 2018 because of pain and right hip decreased range of motion. She underwent a right-sided THA in 2001, when a modular neck implant and femoral stem with proximal titanium porous coating were used (acetabular cup: SPH-CONTACT; femoral stem: F2L Multineck; Lima Corporate, Villanova San Daniele del Friuli, Italy). Early postoperative period was uneventful. In 2012, the patient sustained right-sided trans-acetabular and inferior pubic ramus fractures, which were successfully treated conservatively. Since 2016 she complained about increasing pain in the right groin region and had severely reduced right hip range of motion. Examination in our outpatient clinic showed that her right leg was 2 cm shorter.

**Figure 1:**
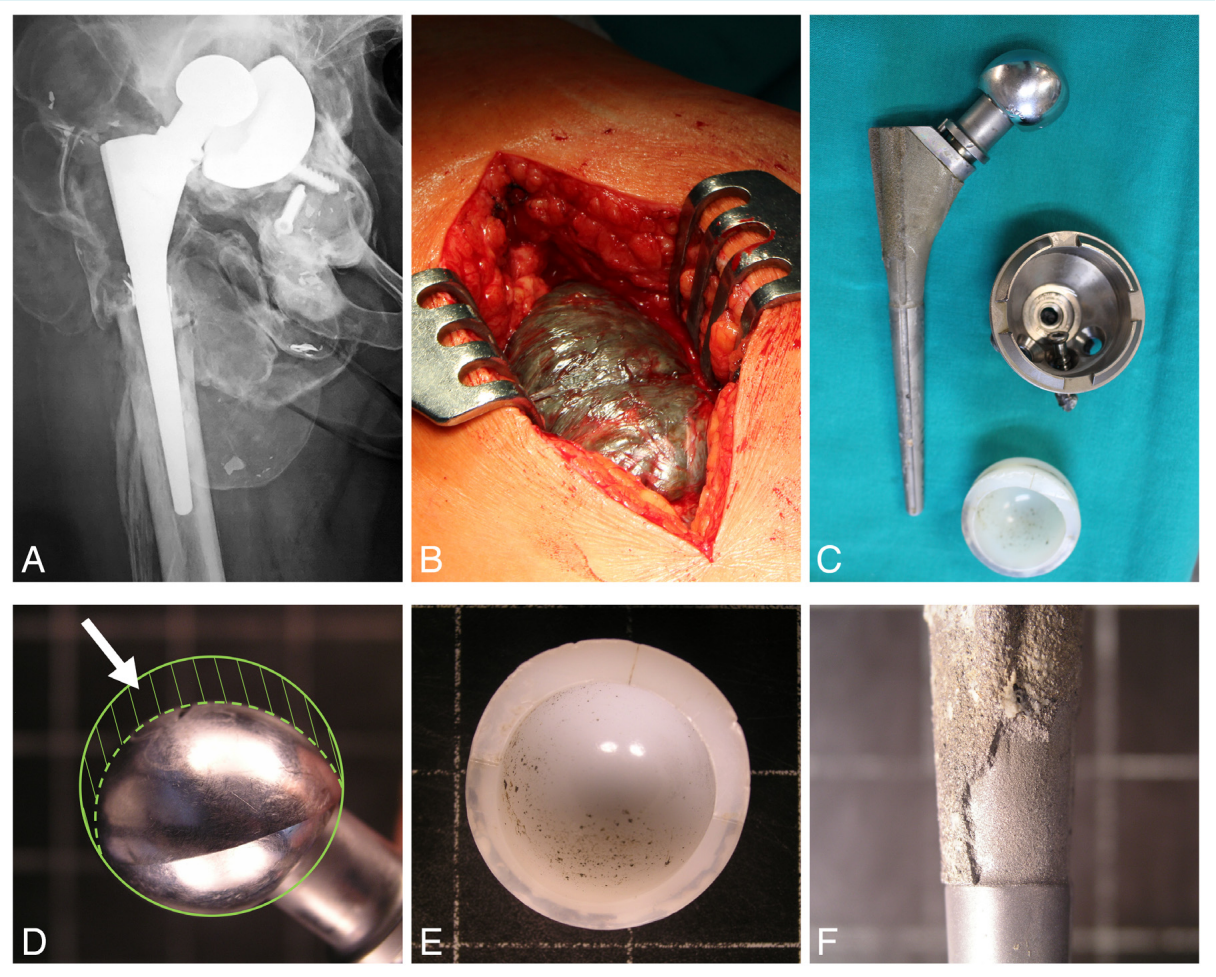
(**A**) Preoperative anteroposterior right hip x-ray showing acetabular cup dislocation, eccentric femoral head wear, “cloudy bubbles,” and pseudotumor formation; (**B**) intraoperative image after a direct lateral approach to the right hip showing extensive metallosis; (**C**) intraoperative image of the extracted endoprosthetic components; (**D**) eccentric wear of the femoral head (posterior view, arrowhead pointing approximately at the worn part of the femoral head); (**E**) metal debris particles embedded in the acetabular polyethylene – most of the particles are embedded in the part of the liner that was not in contact with the worn femoral head; (**F**) damaged titanium porous coating on the extracted femoral stem.

The initial x-ray examination in 2018 showed acetabular cup dislocation, eccentric femoral head wear, “cloudy bubbles” characteristic of metallosis, and pseudotumor formation (Figure 1A). It also showed damage to the titanium porous coating of the femoral stem. A review of the medical records from 2016 revealed femoral head wear *in situ* and damage to the porous stem coating. A revision surgery was indicated, and the patient agreed to the procedure.

During surgery, performed using direct lateral approach, extensive metallosis was observed (Figure 1B, Supplementary Video 1(supplementary )). After thorough debridement and irrigation, all implant components were removed (Figure 1C). The femoral head was gravely worn and elliptically shaped (Figure 1D). The polyethylene liner on the acetabular side had no visible holes or cracks, suggesting there was no direct contact between the femoral head and metal acetabular shell. After endoprosthesis extraction, notable polyethylene liner wear was visible, with metal debris covering the inner surface (Figure 1E). Due to a large acetabular bone defect, it was decided not to proceed with a new acetabular cup implantation. In the postoperative period, a coxofemoral orthosis was applied, and crutches were used for touchdown weight-bearing only. Intraoperatively collected microbiology culture swabs were negative for aerobic and anaerobic microorganisms.

**Figure 2:**
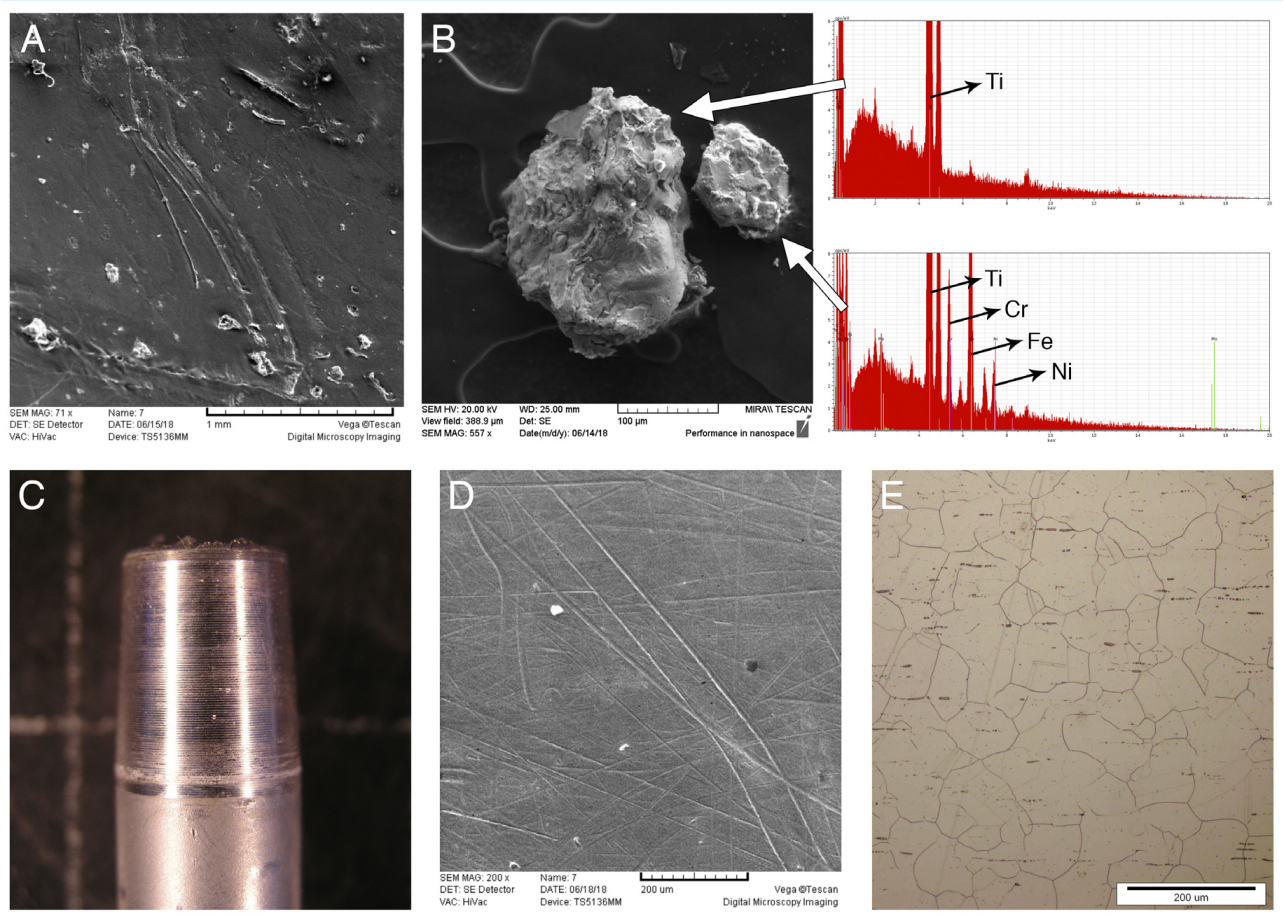
(**A**) Scanning electron microscopy image of the acetabular liner surface that was in contact with the worn femoral head, revealing damage, scratches, indentations, and embedded metal debris particles; (**B**) energy-dispersive spectroscopy image showing that the metal debris embedded in the acetabular liner contains both titanium and austenitic stainless-steel particles; (**C**) the femoral neck at the neck-head junction showing no signs of trunnionosis; (**D**) scanning electron microscopy image of the femoral head surface revealing scratches and damage typical for abrasive mechanical wear; (**E**) microstructure of the femoral head material typical for that kind of material: homogeneous austenitic without any significant defects or other irregularities. Abbreviations: Ti – titanium; Cr – chromium; Fe – iron; Ni – nickel.

Macro- and micro-structure of all extracted components were analyzed in detail at the Faculty of Mechanical Engineering and Naval Architecture of the University of Zagreb. The analysis revealed macroscopic damage to the titanium porous coating of the femoral stem (Figure 1F) and a decreased volume of the femoral head. Metal debris on the acetabular liner was distributed heterogeneously; fewer debris particles were present on the part of the liner adjoining the worn femoral head than on the remaining part of the liner. The surface damage features of the polyethylene were characterized by scanning electron microscopy (SEM) (TESCAN VEGA TS5136LS, TESCAN ORSAY HOLDING a.s., Brno-Kohoutovice, Czech Republic). SEM revealed damage, scratches, indentations, and embedded metal debris particles on the whole acetabular liner surface (Figure 2A). Chemical composition and origin of these particles were determined using energy-dispersive spectroscopy (EDS, OXFORD Instruments, Abingdon, UK), which showed that metal debris consisted of both titanium and stainless-steel particles (Figure 2B).

Femoral head component (AISI 316L stainless-steel) was removed from the femoral neck, and no signs of trunnionosis were observed at the head/neck junction (Figure 2C). To determine the wear mechanisms, the wear tracks on the femoral head surface were analyzed by SEM, and only traces of abrasive wear were found (Figure 2D). The microstructure of the femoral head material was typical for this type of material: homogeneous austenitic without any significant defects or other irregularities (Figure 2E), with microhardness of 145 HV 0.2 (mean value of 5 measurements).

The patient signed the informed consent for publishing the medical data and visual materials.

## Discussion

The main factor limiting long-term THA survival is wear debris production from bearing surfaces (4). Femoral head wear has so far been described only in revision surgery with metal-on-polyethylene bearing after a primary ceramic-on-ceramic THA, when it was caused by ceramic particles embedded in the acetabular liner (3). This is the first report describing femoral head wear in a metal-on-polyethylene primary THA. The wear in our case was caused by abrasion between the femoral head and metal debris from the damaged femoral stem titanium porous coating embedded in the acetabular liner.

Tribocorrosion has been described at the head/neck junction and, more often, at the neck/stem junction (2). In our patient, no tribocorrosion at the head/neck junction was observed. However, it was impossible to evaluate the presence of neck/stem tribocorrosion since we could not separate the neck from the femoral stem (probably due to cold welding) (5).

The main concern of this study was to determine the origin of the metal particles on the polyethylene surface and the type of femoral head wear. The SEM and EDS analysis showed the presence of titanium and austenitic stainless-steel particles (Figure 2B), suggesting that the origin of the titanium particles was the detached titanium porous coating of the femoral stem (Figure 1F). The SEM analysis of the femoral head confirmed that the wear was of the abrasive type and was probably caused by the titanium particles on the polyethylene surface because the hardness of stainless steel femoral head was lower than the titanium hardness described in the literature (145 compared to 200-362 HV 0.2) (6,7).

The only limitation of this study is that microhardness of the porous titanium coating microparticles was not measured due to the method’s unavailability in our setting. Nevertheless, we believe that the comparison with the literature values is justified and that our conclusion is valid.

Our study showed that detached titanium porous coating of the femoral stem can cause the stainless-steel femoral head wear in primary metal-on-polyethylene THA. As soon as such detachment becomes evident on routine follow-up radiographs, revision surgery should be considered to prevent devastating complications described in this report.
